# Work-Related Suicide Exposure, Occupational Burnout, and Coping in Emergency Medical Services Personnel in Poland

**DOI:** 10.3390/ijerph19031156

**Published:** 2022-01-20

**Authors:** Kinga Witczak-Błoszyk, Karolina Krysińska, Karl Andriessen, Jacek Stańdo, Adam Czabański

**Affiliations:** 1 Poznan University of Medical Sciences, 60-512 Poznań, Poland; kns@ump.edu.pl; 2 Melbourne School of Population and Global Health, The University of Melbourne, Melbourne, VIC 3010, Australia; karl.andriessen@unimelb.edu.au; 3 Lodz University of Technology, 90-137 Łódź, Poland; standoj@p.lodz.pl; 4 Department of Social Sciences, Jacob of Paradies University, 66-400 Gorzów Wielkopolski, Poland; aczabanski@interia.pl

**Keywords:** suicide, burnout, coping, stress, workplace, emergency medical service

## Abstract

Work-related suicide exposure may significantly contribute to the risk of burnout in first responders. This study assessed the exposure to suicide, burnout, and coping mechanisms in emergency medical services in Poland, including psychosocial determinants, such as age, gender, and access to psychological support. The level of burnout was assessed using the Link Burnout Questionnaire (LBQ), and coping was measured using the Coping Inventory for Stressful Situations (CISS). Data were analysed using a series of variance analyses and a partial least squares structural equation modelling. The study showed that 98% of emergency medical services personnel reported work-related suicide exposure. The LBQ score indicated symptoms of burnout, in particular relational deterioration, and the CISS showed low levels of emotion-oriented coping. Physicians reported higher levels of psycho-physical exhaustion than paramedics and nurses. Access to psychological support in the workplace was related to lower levels of burnout. Emergency medical services personnel are frequently exposed to suicide, which may be related to the risk of occupational burnout, and coping strategies used in this occupational group are often not optimal. Preventive measures, such as training emergency medical services personnel in regard to effective coping strategies, are needed, and personnel should be encouraged to access mental health services and supports.

## 1. Introduction

Fatal and non-fatal suicidal behaviours remain major public health issues. More than 700,000 people die by suicide every year globally, with a worldwide age-standardized suicide rate of 10.5 per 100,000 people in 2016 [1]; moreover, for every suicide, there are approximately 20 suicide attempts [2]. The impact of suicide can be far reaching [3,4], encompassing the bereaved family members, mental health professionals, and first responders, such as emergency medical services personnel, police officers, and firefighters [5,6]. Studies have shown that first responders, as a professional group, are routinely exposed to suicide [7,8]. For instance, Aldrich and Cerel [9] found that 93% of first responders reported occupational suicide exposure, which was significantly higher than the occupational suicide exposure in other crisis workers (31%) and mental health professionals (56%). Another study found that 70% of ambulance paramedics have been exposed to suicide [10].

Studies show that work-related stress is common in emergency medical services personnel [11,12]. Contributing factors include regular exposure to distressing or traumatic events, non-standard working hours and shift work, excessive workload, physical fatigue, and risk of injury [13,14,15,16]. Working in frequently changing, uncontrolled work environments requires high levels of independence, self-discipline, and quick decision-making, as well as effective teamwork [17]. Paramedics, specialist physicians, specialist nurses, and medical emergency dispatcher professions have “deadly combinations” of job dimensions, e.g., high significance and low autonomy [18]. Additionally, more generic workplace conditions, such as pressure from management, organizational culture, and politics, may contribute to elevated levels of work-related stress in this occupational group [12]. These working conditions may be related to negative mental health impacts, including high levels of post-traumatic stress disorder (PTSD) and other common mental health conditions in emergency medical services personnel [19,20,21,22]. A recent meta-analysis found a 27% prevalence of general psychological distress, 15% of depression or anxiety, and 11% prevalence of PTSD in this occupational group [14]. A UK survey exploring mental health in the emergency services [12] found that 75.8% of ambulance service staff and volunteers had personal experience of mental health problems, mostly depression (56%), anxiety (55%), and PTSD (31%). Furthermore, emergency medical services personnel, and other first responders, may be at a higher suicide risk than other professional groups and the general population [23,24].

Many studies looking at the psychological aftermath of work-related stress in emergency medical services personnel have explored formal coping strategies, such as critical incident stress debriefing [25]. At the same time, informal coping strategies used by emergency medical services personnel may also play a significant role in their ability to cope with work-related stressors [26,27,28]. These include cognitive techniques, such as distancing, avoidance, thinking about the positive benefits of work, professional reflection (i.e., positively reframing a distressing incidence as a learning opportunity), humour, and risky behaviours, such as the use of alcohol and/or drugs [10,26,29]. Another frequent coping mechanism is seeking and utilizing social support, including support from family and friends, work colleagues (especially crew mates), and supervisors/managers [10,29,30].

Elevated levels of work-related stress may significantly contribute to the risk of burnout in emergency medical services personnel [31,32]. Burnout, or a prolonged response to chronic emotional and interpersonal stressors on the job, is defined by three key dimensions: overwhelming exhaustion, cynicism and detachment from the job, and inefficacy and lack of accomplishment [33]. A systematic review of prevalence studies reported that between 16% and 56% of paramedics suffer from burnout; the disparity possibly results from the complexity of burnout syndrome itself as well as the heterogeneity of measurement tools [34]. Possible correlates of burnout in emergency medical services personnel include gender, age, education level, work type and location, years of employment/experience, work overload, emotional problems at work, and physical health limitations [34].

Despite the importance of the subject, there is only limited knowledge on the prevalence and the psychological aftermath of suicide exposure in emergency medical services personnel [7,9]. The current study aimed to address this gap by assessing exposure to suicide in emergency medical services in Poland (research question 1, Q1) and by measuring levels of burnout and informal coping mechanisms in this occupational group (research questions 2 and 3, Q2 and Q3, respectively).

The study also aimed to explore the psychosocial determinants of burnout and coping mechanisms in this occupational group, such as marital status and type of occupation (such as paramedic, nurse, or physician), level and timing of suicide exposure, the availability of and access to psychological support at work and other support systems, demographic (age and gender) and work-related characteristics (work seniority and workload). In this regard, we next explored the following five research questions: Q4. Are there differences in the level and timing of suicide exposure depending on the work setting?; Q5. Are there differences between the levels of burnout and informal coping style depending on the marital status and occupational group?; Q6. Are burnout and informal coping mechanisms related to the level of suicide exposure (Model 1)?; Q7. Are burnout and coping related to access to professional psychological support and other workplace support programs (Model 2)?; and Q8. Are burnout, and coping related to the age, gender, years in employment or workload (Model 3)?

## 2. Materials and Methods

### 2.1. Participants

The emergency medical services in Poland have a duty to assist a person in a state of health emergency [16,35]. The emergency medical services units encompass hospital emergency departments and emergency medical teams, specialist hospital wards, emergency rooms, and rescue services (such as Mountain Volunteer Search and Rescue). The emergency medical services personnel in Poland include paramedics, specialist physicians, specialist nurses, and medical emergency dispatchers. Study participants were recruited in regional and local emergency medical services units, emergency departments, and air ambulance stations in the Wielkopolskie Voivodeship. Eligible participants were active emergency medical services personnel in the Wielkopolskie Voivodeship, who were aged between 25 and 60 years.

The Bioethics Commission of Poznań University of Medical Sciences approved the study (166/16, 4 February 2016). All participants provided informed consent, and the study was conducted between June 2018 and May 2019.

### 2.2. Measures

A 30-item custom-designed questionnaire included demographic questions (age, gender, place of residence, marital status, profession, years in employment, workload, and work setting), questions about workplace suicide exposure (Q1) (frequency, timing, distress), the availability of workplace psychological support, and the availability of informal social support (e.g., family, friends, colleagues, mental health professionals).

The Link Burnout Questionnaire (LBQ) is a 24-item self-report questionnaire designed to measure burnout syndrome in healthcare professionals [36,37,38]. LBQ items are rated on a 6-point Likert scale ranging from (1) (Never) to (6) (Every day). The LBQ measures four dimensions of burnout: psycho-physical exhaustion (6 items); relational deterioration (quality of relationships with patients/clients; 6 items); professional inefficacy (a sense of decreased professional competency; 6 items); and disillusion (existential expectations; 6 items) [39].

The Coping Inventory for Stressful Situations (CISS) is a 48-item self-report inventory designed to measure strategies used to cope with stress [40,41]. CISS statements are rated on a 5-point Likert-type rating scale ranging from (1) (Not at all) to (5) (Very much). The CISS measures three dimensions, which determine the preferred informal coping style: task-oriented (16 items), emotion-oriented (16 items), and avoidance coping (16 items). Avoidance coping is divided into two subscales: distraction (engaging in replacement activities; 8 items) and social diversion (seeking social contact; 8 items) [42].

### 2.3. Data Analysis

The level of burnout (Q2) was assessed using LBQ scores, based on norms in the Polish population, designed to relate test results to the occupational group of physicians [36,37]. The informal coping style (Q3) was assessed using CISS scores based on norms for the age group between 25 and 54 in the Polish population [41]. A series of variance analyses for independent samples assessed differences in the level and timing of suicide exposure depending on the work setting (Q4), and whether marital status and occupational group differentiated between levels of burnout and informal coping style (Q5).

Partial least squares (PLS) structural equation modelling was performed using WarpPLS 6.0 software [43] to explore the other three study research questions through verifying the predictive properties of the three models conceptualized based on the literature [10,25,33]. PLS structural equation modelling is a confirmatory statistical analysis with an aim to maximize the amount of explained covariance of dependent variables by means of a number of predictors. The first step is the formation of latent variables using confirmatory factor analysis performed on manifest variables, i.e., questionnaire items [43]. The construction of a measurement model of the structural model results in formed variables, which are ready for multidimensional path analysis. The criterion for evaluation of the measurement model is the assessment of convergence of empirical data with the variable measurement model put forth by the researcher. In the second step, multivariate regression analyses are performed in the course of iteration to maximize the prediction of dependent variables. This contributes to the verification of the theoretical model conceptualized by the researcher and expressed in the empirical path model. A path diagram of the structural model provides feedback on the significance of the relationship between predictors and dependent variables. The most important criterion for evaluation of the path model is the generalized predictive power of dependent variables.

Our analysis used the PTH1 algorithm, which relies on the information that the variable measurement model is reflective. Consequently, the model is calculated with regard to the fact that the latent variable influences the variance of manifest variables, which are measured with an inherent measurement error [44]. Standard errors and statistical significance were estimated by means of the Stable3 method, used for calculating statistical significance and proposed by the software developer [45]. In this method, errors are calculated in the process of exponential smoothing and not in the process of bootstrapping [46]. Linear relationships between variables were predicted for the analysis.

## 3. Results

### 3.1. Demographic Profile and Suicide Exposure (Q1)

The study sample (*N* = 411) comprised 249 males (60.6%) and 162 females (39.4%), and most respondents (74%) were in the 25–40 age group. Paramedics were the largest professional group (71.8%), followed by nurses (15.8%), medical doctors (9.0%), and other medical professionals, such as radiologists (3.4%). Respondents worked mostly in car ambulance teams (62.3%), emergency departments (28.9%) and in air ambulance teams (8.7%). Most (98%) of the study respondents reported occupational exposure to suicide (Q1), and 43% experienced this as “distressing”.

Almost half of the respondents reported that professional psychological support was available at their workplace (47%) and/or they had access to professional psychological support elsewhere (44.5%), and 7.5% reported access to other forms of psychological support. The remaining respondents either had no access or were unsure. Most respondents were willing to seek psychological help (56.4%), whereas one in four was unwilling (24.3%) and one in five was unsure (19.2%). Work colleagues (71.5%), family members (39.7%), and/or friends (21.7%) were the most frequently used sources of informal support in the aftermath of suicide exposure; however, one in four respondents did not seek any support following suicide exposure (18.5%). Talking to work colleagues was also the most frequently reported informal coping strategy after suicide exposure (62.4%), followed by physical activity (43.7%), and drug/alcohol use (30.2%). Table 1 details the demographic characteristics of the respondents.

### 3.2. Q2 and Q3: Burnout and Coping

The LBQ score indicated that respondents were liable to occupational burnout and were already exhibiting symptoms of burnout, in particular, relational deterioration (raw score 22.59, 90% CI 18.59–26.59) (Table 2). There were moderate levels of psycho-physical exhaustion, professional inefficacy, and disillusion in the study sample. The CISS showed that emotion-oriented coping (raw score 35.49, 90% CI 29.49–41.49) was the least frequently used informal coping style in the study sample. The use of task-oriented and avoidance coping, either as distraction or social diversion, was moderate.

### 3.3. Q4: Work Setting, Suicide Exposure, Time of Suicide Exposure, and Level of Suicide Exposure

There was no statistically significant difference between work settings (car ambulance team, emergency department, air ambulance team) in regard to having been exposed to suicide in the workplace (*F*(2,280) = 1.43; *p* > 0.05, η^2^ = 0.01) or the level of suicide exposure (*F*(2,280) = 2.20; *p* > 0.05, η^2^ = 0.02). Respondents employed in an ambulance team were exposed to suicide significantly earlier in their career than those working in an air ambulance (*F*(2,280) = 3.55; *p* < 0.05, η^2^ = 0.03).

### 3.4. Q5: Occupation, Marital Status, Burnout, and Coping

There were statistically significant differences between different professional groups (paramedic, nurse, physician) regarding levels of burnout and preferred informal coping styles. Specifically, psycho-physical exhaustion was higher in physicians than other groups (*F*(3,384) = 4.18; *p* < 0.05, η^2^ = 0.02), emotion-oriented coping was higher among physicians and nurses than paramedics (*F*(3,384) = 6.87; *p* < 0.01, η^2^ = 0.04), and avoidance coping, distraction, and social diversion was more prevalent among nurses than paramedics and physicians (*F*(3,384) = 8.14; *p* < 0.01, η^2^ = 0.04; *F*(3,384) = 4.50; *p* < 0.05, η^2^ = 0.02; and *F*(3,384) = 7.65; *p* < 0.01, η^2^ = 0.04; respectively).

There were differences between marital status groups (married, with partner, single, divorced, widowed) regarding levels of burnout and preferred informal coping styles. The highest intensity of disillusionment was reported by divorced respondents, lower by married respondents, and the lowest level of disillusionment was found among respondents in partner relationships and single (*F* (3,384) = 5.52; *p* < 0.01, η^2^ = 0.04). Task-oriented coping and distraction were most frequently used by those in partner relationships (*F* (3,384) = 2.97; *p* < 0.05, η^2^ = 0.02; *F* (3,384) = 2.93; *p* < 0.05, η^2^ = 0.02; respectively), whereas other subgroups of respondents had similar lower levels.

### 3.5. Q6 (Model 1): Suicide Exposure, Burnout and Coping

The analysis of the measurement model quality assessment coefficient and data adjustment indicated that there was no significant collinearity among the predictors in the path model AVIF = 1.12 and there was very good data adjustment to the measurement model SRMR = 0.12, SMAR = 0.09. Variable indicators were significantly associated with latent variables χ^2^ = 53.4; *p* < 0.001. The analysis of the general predictive power of the model revealed that it had moderate predictive power GoF = 0.19. (Table S1; Supplementary Materials). A series of analyses indicated that the measurements performed in the study had moderate/high reliability α = 0.64–0.91 and high average variance extracted, AVE = 0.30–0.53 (Table S2; Supplementary Materials). Analysis of the path coefficients indicated that suicide exposure was related to relational deterioration (β = 0.13, *p* < 0.01). Later suicide exposure was related to higher professional inefficacy (β = 0.18, *p* < 0.001) and higher levels of emotion-oriented coping (β = 0.12, *p* = 0.01).

Higher suicide exposure was related to higher levels of three aspects of burnout: psycho-physical exhaustion (β = 0.13, *p* < 0.01), relational deterioration (β = 0.42, *p* < 0.001), and disillusion (β = 0.22, *p* < 0.001), as well as a higher frequency of task-oriented coping (β = 0.14, *p* < 0.01). Higher suicide exposure was also related to lower levels of avoidance (β = 0.20, *p* < 0.01), both through distraction (β = −0.16, *p* < 0.001) and social diversion (β = −0.19, *p* < 0.001). The detailed results are presented in Table 3.

### 3.6. Q7 (Model 2): Access to Professional Psychological Support and Other Workplace Support Programs, Burnout, and Coping

Analysis of the measurement model quality assessment coefficient and data adjustment indicated that there was no significant collinearity among the predictors in the path model, AVIF = 1.75. The analysis indicated very good data adjustment to the measurement model and variable indicators were significantly associated with latent variables. The analysis of the general predictive power of the model revealed that it had moderate predictive power, GoF = 0.12. (Table S3; Supplementary Materials). Measurements performed in the study had moderate/high reliability α = 0.64–0.91 and high average variance extracted, AVE = 0.30–0.53 (Table S4; Supplementary Materials).

Analysis of the path coefficients indicated that the availability of professional psychological support at the workplace was related to lower levels of relational deterioration (β = −0.12, *p* < 0.01) and disillusionment (β = −0.12, *p* < 0.01), and higher frequencies of task-oriented coping (β = 0.14, *p* < 0.01), avoidance (β = 0.12, *p* < 0.01), and social diversion (β = 0.13, *p* < 0.01). Other access to psychological support was related to lower levels of psycho-physical exhaustion (β = −0.10, *p* < 0.01) and lower relational deterioration (β = −0.09, *p* < 0.05). Furthermore, the availability of other workplace support programs was related to lower relational deterioration (β = −0.13, *p* < 0.01) and lower disillusion (β = 0.10, *p* < 0.05). This variable was not related to any aspects of informal coping styles. The detailed results are presented in Table 4.

### 3.7. Q8 (Model 3): Age, Gender, Years in Employment, Workload, Burnout, and Coping

Analysis of the measurement model quality assessment coefficient and data adjustment indicated that there was no significant collinearity among the predictors in the path model, AVIF = 2.72. The analysis indicated very good data adjustment to the measurement model, SRMR = 0.12, SMAR = 0.09, and variable indicators were significantly associated with latent variables, χ^2^ = 56.73; *p* < 0.001. Analysis of the general predictive power of the model revealed that it had moderate predictive power, GoF = 0.19 (Table S5; Supplementary Materials). Measurements performed in the study had moderate/high reliability, α = 0.64–0.91, and high average variance extracted, AVE = 0.30–0.53. (Table S6; Supplementary Materials).

The analysis of path coefficients indicated that older age was related to higher psycho-physical exhaustion (β = 0.19, *p* < 0.001), professional inefficacy (β = 0.18, *p* < 0.001) and disillusion (β = 0.12, *p* < 0.01), and lower relational deterioration (β = −0.31, *p* < 0.001). Older age was also related to the less frequent use of task-oriented coping (β = −0.11, *p* < 0.05), avoidance (β = −0.18, *p* < 0.001), and using both distraction (β = −0.16, *p* < 0.001) and social diversion (β = −0.15, *p* < 0.01). There were no differences between men and women regarding levels of burnout, except for relational deterioration (β = −0.14, *p* < 0.05). Women used emotion-oriented coping (β = 0.22, *p* < 0.001), avoidance (β = 0.17, *p* < 0.001), and both distraction (β = 0.11, *p* < 0.05) and social diversion (β = 0.17, *p* < 0.01) more often than men.

More years in employment were related to higher levels of relational deterioration (β = 0.10, *p* < 0.05) and more frequent task-oriented coping (β = 0.13, *p* < 0.01), as well as lower professional inefficacy (β = −0.18, *p* < 0.001) and less emotion-oriented coping (β = −0.09, *p* < 0.05). Higher workload was related to lower professional inefficacy (β = −0.11, *p* < 0.05), higher relational deterioration (β = 0.48, *p* < 0.001) and higher disillusion (β = 0.09, *p* < 0.05), as well as the more frequent use of task-oriented coping (β = 0.18, *p* < 0.001). The detailed results are presented in Table 5.

## 4. Discussion

To the best of our knowledge, this is this is the first study exploring occupational burn-out and coping in emergency medical services personnel exposed to suicide. Study results regarding the first research question (Q1) showed that almost all emergency medical services personnel (98.1%) have been exposed to suicide in their professional career, and almost half (43.1%) experienced this event as “distressing”. Previous studies on suicide exposure in emergency medical services personnel found similarly high levels of exposure: 93% in a study by Aldrich and Cerel [9], and 70% in a study by Regehr and colleagues [10]. In regard to the fourth research question (Q4), although there were no differences in the level of exposure between emergency medical services personnel working in the three work settings included in the study, i.e., car ambulance, emergency department, and air ambulance; car ambulance personnel were exposed to suicide earlier in their career than those working in the other settings. A lack of previous studies on the differences in suicide exposure in different emergency medical services settings precludes comparisons

The results of our study (Q2) confirm that emergency medical services personnel are an occupational group at risk of occupational burnout [47,48]. Our respondents reported moderate to high levels of burnout, in particular regarding relational deterioration, and higher levels of suicide exposure were related to higher scores across the domains of burnout. To the best of our knowledge, no previous studies have explored burnout specifically in relation to suicide exposure in emergency medical services personnel; nonetheless, trauma-related emergencies are a well-known risk factor for burnout in this occupational group [34]. In regard to research question Q5, our results indicate that levels of burnout and coping style could be related to marital status in emergency medical services personnel. Respondents who were divorced reported the highest level of disappointment with life; this aspect of burnout was lower in respondents who were married, in partner relationships and single, and task-oriented coping and distraction was most frequently used by those in partner relationships. Similar observations were reported in previous studies [49], which indicates the important role family support may play in coping with the negative effects of work-related stress [30,50]. Furthermore, regarding the eighth research question (Q8), our study shows that the levels of burnout increase with age, years in employment, and workload, which reflects previous research outcomes [49,50,51,52,53,54,55].

In our study, emotion-oriented coping was the least frequently used informal coping style among the emergency medical services personnel (Q3). This style, marked by a focus on oneself and one’s emotions when faced with a stressor, is considered the least adaptive and the least effective [56]. Although task-oriented coping is considered to be the most effective [56], we found that the emergency medical services personnel were not always able to overcome difficulties in stressful situations: they may abandon efforts to solve a problem and do not always attempt to change their situation. Furthermore, regarding the sixth and eighth research questions (Q6 and Q8), higher suicide exposure and higher workload were related to higher scores for task-oriented coping, whereas the later the suicide exposure, the lower the frequency of task-oriented coping.

Our analysis related to the fifth research question (Q5) showed differences between the three occupational groups in our study (paramedics, nurses, and physicians) regarding the levels of burnout and the preferred coping style. In particular, physicians reported higher levels of psycho-physical exhaustion and often used an emotion-oriented coping style, a result reported in previous studies [57,58]. This may be related to the fact that physicians are more frequently exposed to the strong emotional reactions of patients and their families when informing them about adverse medical prognosis or death [47], and/or indicate that some ED clinicians may be inadequately prepared to cope with occupational stressors [59].

Regarding the eighth research question (Q8), older emergency medical services personnel relied less on coping with stress through a task-oriented style or avoidance. These results suggest that the ability to cope with stress through an attempt to solve or limit the impact of the stressful situations may increase with work experience [27], but necessarily with age. The emergency medical services personnel also exhibited moderate use of an avoidant coping style, either as a distraction or social diversion, indicating that they tend to avoid confrontation with difficult situations. These results suggest that emergency medical services personnel could benefit from learning to use a more adaptive task-oriented style of coping with stressful situations [30,60].

Our analysis related to the eighth research question (Q8) showed no gender differences in the levels of burnout (except for relational deterioration), although men relied less on coping with stress through a emotion-oriented style or avoidance, either through distraction or social diversion. Reviews of the literature have flagged the complex relationship between gender and different aspects of burnout in paramedics (e.g., patient- versus personal-related burnout) [34] and gender and informal coping strategies [26]. Further studies are needed to better understand this relationship in the emergency medical services personnel in the context of suicide exposure.

Regarding the seventh research question (Q7), our study indicated the significant role of having access to professional mental health support, including access to a psychologist in the workplace, in reducing the levels of burnout in emergency medical services personnel. This finding is in line with studies showing a significant reverse correlation between the availability of psychological support and occupational burnout [61]. Nonetheless, only approximately half of respondents in our study had access to a psychologist or other psychological support in their workplace and/or were willing to seek psychological help, and the availability of psychological support was not related to the use of more effective coping strategies, such as task-oriented coping. This signals a need to raise awareness and facilitate access to psychological support at work and for increased opportunities to learn effective ways to compensate stress and prevent symptoms of burnout [62,63].

### Limitations

The cross-sectional design is a limitation of our study because it precludes defining direct causation between the variables included in the analysis. The term “work-related suicide exposure” has been conceptualized in terms of frequencies and/or dichotomized “Yes/No” types of exposure, which might have oversimplified this complex variable [5,9]. The study was conducted before the start of the COVID-19 pandemic in early 2020, and it is possible that the levels of burnout and coping strategies of the emergency medical services personnel have been affected by the changed working conditions [64], as well as changes in the prevalence of suicide in Poland [65]. Furthermore, the study focused on suicide exposure and did not compare this work-related stressor to other occupational risk in emergency medical services personnel [66].

## 5. Conclusions

Our study has supported the observation that emergency medical services personnel are frequently exposed to suicide as part of their professional activities. Suicide exposure is related to higher levels of occupational burnout; however, the informal coping strategies used by the emergency medical services personnel are often not optimal. These findings emphasize the need to continue implementing preventive measures, such as training emergency medical services personnel in regard to effective coping strategies and encouraging use of mental health services and supports. Additionally, future studies are indispensable in order to deepen our understanding of the frequency and impact of occupational suicide exposure in first responders, which may be particularly important in the times of the COVID-19 pandemic.

## Figures and Tables

**Table 1:** Demographic characteristics (*N* = 411).

Variable		*N* (%)
Gender	Male	249 (60.6%)
	Female	162 (39.4%)
Age	<25	35 (8.5%)
	25–30	127 (30.9%)
	31–35	110 (26.8%)
	36–40	67 (16.3%)
	41–45	38 (9.2%)
	46–50	22 (5.4%)
	>50	12 (2.9%)
Relationship status	Married	201 (48.9%)
	With a partner	57 (13.9%)
	Single	114 (27.7%)
	Divorced	36 (8.8%)
	Widowed	3 (0.7%)
Professional group	Paramedics	295 (71.8%)
	Nurses	65 (15.8%)
	Medical doctors	37 (9.0%)
	Other medical professionals	14 (3.4%)
Workplace setting	Ambulance team	207 (62.3%)
	Emergency department	96 (28.9%)
	Air ambulance	29 (8.7%)
Type of employment	>Full-time	301 (73.2%)
	Full-time	96 (23.4%)
	<Full-time	14 (3.4%)
Location	>100,000 inhabitants	149 (36.3%)
	50,000–100,000 inhabitants	58 (14.1%)
	10,000–50,000 inhabitants	83 (20.2%)
	Up to 10,000 inhabitants	41 (10%)
	<10,000 inhabitants	80 (19.5%)
Suicide exposure	Yes	403 (98.1%)
	First week of employment	48 (16.3%)
	First month of employment	71 (24.1%)
	First three months of employment	54 (18.4%)
	First six months of employment	31 (10.5%)
	First year of employment	57 (19.4%)
	Other	33 (11.2%)
	No	8 (1.9%)
Available support -Professional psychological support at workplace	Yes	193 (47%)
	No	166 (40.4%)
	Unsure	52 (12.7%)
-Access to psychological support elsewhere	Yes	183 (44.5%)
	No	117 (28.5 %)
	Unsure	111 (27%)
-Other professional support at workplace	Yes	31 (7.5 %)
	No	311 (75.7%)
	Unsure	69 (16.8%)
-Financial support	Yes	59 (14.4%)
	No	330 (80%)
	Unsure	22 (5.4%)
Suicide intervention experienced as distressing	Yes	177 (43.1%)
	No	136 (33.1%)
	Unsure	98 (23.8%)
Readiness/willingness to seek psychological help	Yes	232 (56.4%)
	No	100 (24.3%)
	Unsure	79 (19.2%)
Source of support following suicide exposure ^1^	Work colleagues	294 (71.5%)
	Family members	163 (39.7%)
	Friends	89 (21.7%)
	Acquittances	36 (8.8%)
	Psychologist	20 (4.9%)
	Clergy	12 (2.9%)
	Nobody	76 (18.5%)
Informal coping following suicide exposure ^1^	Talking to work colleagues	256 (62.4%)
	Physical activity	179 (43.7%)
	Drugs/alcohol	124 (30.2%)
	Cultural activities	67 (16.3%)
	Prayer	41 (10%)
	Talking to a psychologist	13 (3.2%)
	Talking to clergy	11 (2.7%)

^1^ Does not add up to 100% because multiple answers were possible.

**Table 2:** Scores of burnout (Link Burnout Questionnaire) and coping (Coping Inventory for Stressful Situations).

Subscale	Raw Score (With 90% Confidence Interval)	Sten	Score
**Burnout**			
Psycho-physical exhaustion	14.56 (19.56) 24.56	5 (7) 8	Moderate
Relational deterioration	18.59 (22.59) 26.59	7 (9) 10	High
Professional inefficacy	10.00 (14.00) 18.00	4 (6) 8	Moderate
Disillusion	12.98 (16.98) 20.98	6 (7) 8	Moderate
**Coping**			
Task-oriented coping	50.76 (56.76) 62.76	4 (5) 7	Moderate
Emotion-oriented coping	29.49 (35.49) 41.49	2 (3) 5	Low/ Moderate
Avoidance coping	32.23 (40.23) 48.23	3 (5) 7	Moderate
Distraction	12.12 (17.12) 22.12	3 (5) 7	Moderate
Social diversion	11.38 (15.38) 19.38	3 (5) 7	Moderate

**Table 3:** Estimation of structural model path coefficients (Model 1: Suicide Exposure, Burnout, and Coping).

Predictor	Dependent Variable	β	*p*
**Suicide exposure**	Psycho-physical exhaustion	−0.06	0.129
	**Relational deterioration**	**0.13**	**0.004**
	Professional inefficacy	−0.02	0.358
	Disillusion	−0.03	0.270
	Task-oriented coping	0.00	0.472
	Emotion-oriented coping	−0.08	0.064
	Avoidance coping	−0.05	0.148
	Distraction	−0.06	0.110
	Social diversion	−0.02	0.356
**Time of suicide exposure**	Psycho-physical exhaustion	0.04	0.218
	Relational deterioration	−0.03	0.246
	**Professional inefficacy**	**0.18**	**<0.001**
	Disillusion	−0.01	0.400
	Task-oriented coping	−0.08	0.054
	**Emotion-oriented coping**	**0.12**	**0.010**
	Avoidance coping	0.04	0.237
	Distraction	0.03	0.270
	Social diversion	0.01	0.428
**Level of suicide exposure**	**Psycho-physical exhaustion**	**0.13**	**0.005**
	**Relational deterioration**	**0.42**	**<0.001**
	Professional inefficacy	0.08	0.051
	**Disillusion**	**0.22**	**<0.001**
	**Task-oriented coping**	**0.14**	**0.002**
	Emotion-oriented coping	−0.07	0.088
	**Avoidance coping**	**−0.20**	**<0.001**
	**Distraction**	**−0.16**	**<0.001**
	**Social diversion**	**−0.19**	**<0.001**

Values in bold are statistically significant.

**Table 4:** Estimation of structural model path coefficients (Model 2: Access to Professional Support, Burnout, and Coping).

Predictor	Dependent Variable	β	*p*
**Psychological support available at workplace**	Psycho-physical exhaustion	−0.06	0.109
	**Relational deterioration**	**−0.12**	**0.007**
	Professional inefficacy	−0.03	0.295
	**Disillusion**	**−0.12**	**0.008**
	**Task-oriented coping**	**0.14**	**0.003**
	Emotion-oriented coping	0.08	0.061
	**Avoidance coping**	**0.12**	**0.009**
	Distraction	0.06	0.121
	**Social diversion**	**0.13**	**0.005**
**Other access to psychological support**	**Psycho-physical exhaustion**	**−0.10**	**0.020**
	**Relational deterioration**	**−0.09**	**0.042**
	Professional inefficacy	0.01	0.427
	Disillusion	−0.04	0.205
	Task-oriented coping	−0.04	0.219
	Emotion-oriented coping	0.00	0.500
	Avoidance coping	−0.01	0.458
	Distraction	−0.03	0.267
	Social diversion	0.04	0.201
**Other workplace support system**	Psycho-physical exhaustion	−0.01	0.448
	**Relational deterioration**	**−0.13**	**0.004**
	Professional inefficacy	0.02	0.358
	**Disillusion**	**−0.10**	**0.027**
	Task-oriented coping	0.03	0.254
	Emotion-oriented coping	0.01	0.449
	Avoidance coping	0.00	0.500
	Distraction	0.01	0.398
	Social diversion	0.00	0.483

Values in bold are statistically significant.

**Table 5:** Estimation of structural model path coefficients (Model 3: Age, Gender, Years in Employment, Workload).

Predictor	Dependent Variable	β	*p*
**Age**	**Psycho-physical exhaustion**	**0.19**	**<0.001**
	**Relational deterioration**	**−0.31**	**<0.001**
	**Professional inefficacy**	**0.18**	**<0.001**
	**Disillusion**	**0.12**	**0.007**
	**Task-oriented coping**	**−0.11**	**0.017**
	Emotion-oriented coping	0.02	0.367
	**Avoidance coping**	**−0.18**	**<0.001**
	**Distraction**	**−0.16**	**<0.001**
	**Social diversion**	**−0.15**	**0.002**
**Gender**	Psycho-physical exhaustion	−0.01	0.420
	**Relational deterioration**	**−0.14**	**0.002**
	Professional inefficacy	−0.01	0.397
	Disillusion	−0.06	0.102
	Task-oriented coping	0.06	0.124
	**Emotion-oriented coping**	**0.22**	**<0.001**
	**Avoidance coping**	**0.17**	**<0.001**
	**Distraction**	**0.11**	**0.012**
	**Social diversion**	**0.17**	**<0.001**
**Years in employment**	Psycho-physical exhaustion	0.01	0.440
	**Relational deterioration**	**0.10**	**0.028**
	**Professional inefficacy**	**−0.18**	**<0.001**
	Disillusion	0.03	0.294
	**Task-oriented coping**	**0.13**	**0.004**
	**Emotion-oriented coping**	**−0.09**	**0.037**
	Avoidance coping	−0.03	0.258
	Distraction	0.01	0.451
	Social diversion	−0.01	0.414
**Workload**	Psycho-physical exhaustion	−0.06	0.129
	**Relational deterioration**	**0.48**	**<0.001**
	**Professional inefficacy**	**−0.11**	**0.012**
	**Disillusion**	**0.09**	**0.033**
	**Task-oriented coping**	**0.18**	**<0.001**
	Emotion-oriented coping	−0.07	0.075
	Avoidance coping	0.05	0.165
	Distraction	0.04	0.211
	Social diversion	0.06	0.112

Values in bold are statistically significant.

## Data Availability

Data are available upon reasonable request from the corresponding author.

## Supplementary Materials

The following supporting information can be downloaded at: https://www.mdpi.com/article/10.3390/ijerph19031156/s1, Table S1: Model fit, Table S2: Measurement accuracy of the scales used and the explained measurement variance, Table S3: Model fit, Table S4: Measurement accuracy of the scales used and the explained measurement variance, Table S5: Model fit, Table S6: Measurement accuracy of the scales used and the explained measurement variance.

## References

[1] World Health Organization (2021). Suicide in the World: Global Health Estimates.

[2] World Health Organization (2016). Preventing Suicide: A Global Imperative.

[3] Andriessen K., Krysinska K., Grad O. (2017). Postvention in Action: The International Handbook of Suicide Bereavement Support.

[4] Andriessen K., Rahman B., Draper B., Dudley M., Mitchell P.B. (2017). Prevalence of exposure to suicide: A meta-analysis of population-based studies. J. Psychiatr. Res..

[5] Cerel J., McIntosh J.L., Neimeyer R.A., Maple M., Marshall D. (2014). The continuum of “survivorship”: Definitional issues in the aftermath of suicide. Suicide Life Threat. Behav..

[6] Nelson P.A., Cordingley L., Kapur N., Chew-Graham C.A., Shaw J., Smith S., McGale B., McDonnell S. (2020). ‘We’re the First Port of Call’—Perspectives of ambulance staff on responding to deaths by suicide: A qualitative study. Front. Psychol..

[7] Lyra R.L.D., McKenzie S.K., Every-Palmer S., Jenkin G. (2021). Occupational exposure to suicide: A review of research on the experiences of mental health professionals and first responders. PLoS ONE.

[8] Norton K. (2017). Responding to a suicide death: The role of first responders. Death Stud..

[9] Aldrich R.S., Cerel J. (2020). Occupational suicide exposure and impact on mental health: Examining differences across helping professions. Omega.

[10] Regehr C., Goldberg G., Hughes J. (2002). Exposure to human tragedy, empathy, and trauma in ambulance paramedics. Am. J. Orthopsychiatry.

[11] Lelonek B., Kołodziej M., Chmielewski J., Merecz-Kot D., Szpringer M. (2016). Stres w zawodzie Ratownika Medycznego [Stress in paramedics]. Zawodowe i Społeczne Problemy Ochrony Zdrowia [Occupational and Social Problems in Public Health].

[12] MIND (2016). Mental Health in the Emergency Services Our 2019 Survey Results—Ambulance Service. https://www.mind.org.uk/media-a/4847/2019-survey-ambulance-service-summary.pdf.

[13] Kulczycka K., Grzegorczyk-Puzio E., Strychno E., Piasecki J., Strach R. (2016). Wpływ pracy na samopoczucie ratowników medycznych [Effect of work on general wellbeing of paramedics]. Medycyna Ogólna Nauki O Zdrowiu.

[14] Petrie K., Milligan-Saville J., Gayed A., Deady M., Phelps A., Dell L., Forbes D., Bryant R.A., Calvo R.A., Glozier N. (2018). Prevalence of PTSD and common mental disorders amongst ambulance personnel: A systematic review and meta-analysis. Soc. Psychiatry Psychiatr. Epidemiol..

[15] Courtney J.A., Francis A.J., Paxton S.J. (2010). Caring for the carers: Fatigue, sleep, and mental health in Australian paramedic shiftworkers. Aust. N. Z. J. Organ. Psychol..

[16] Wnukowski K., Kopański Z., Sianos G. (2015). Specyfika pracy ratownika medycznego [The nature of work in medical rescue]. J. Clin. Healthc..

[17] Fedorczuk W., Pawlas R. (2011). Ryzyko zawodowe w pracy ratownika medycznego. Hygeia Public Health.

[18] Johns G. (2010). Some unintended consequences of job design. J. Organ. Behav..

[19] Berger W., Coutinho E.S.F., Figueira I., Marques-Portella C., Luz M.P., Neylan T.C., Marmar C.R., Mendlowicz M.V. (2012). Rescuers at risk: A systematic review and meta-regression analysis of the worldwide current prevalence and correlates of PTSD in rescue workers. Soc. Psychiatry Psychiatr. Epidemiol..

[20] Jones S. (2017). Describing the mental health profile of first responders: A systematic review. J. Am. Psychiatr. Nurses Assoc..

[21] Kucmin T., Kucmin A., Turska D., Turski A., Nogalski A. (2018). Style radzenia sobie ze stresem i dyspozycyjny optymizm jako predyktory nasilenia objawów PTSD w grupie ratowników medycznych [Coping styles and dispositional optimism as predictors of post-traumatic stress disorder (PTSD) symptoms intensity in paramedics]. Psychiatr. Pol..

[22] Wagner S.L., White N., Regehr C., White M., Alden L.E., Buys N., Carey M.G., Corneil W., Fyfe T., Matthews L.R. (2020). Ambulance personnel: Systematic review of mental health symptoms. Traumatology.

[23] Carleton R.N., Afifi T.O., Turner S., Taillieu T., LeBouthillier D.M., Duranceau S., Asmundson G.J.G. (2018). Suicidal ideation, plans, and attempts among public safety personnel in Canada. Can. Psychol..

[24] Stanley H.I., Hom M.A., Joiner T.E. (2016). A systematic review of suicidal thoughts and behaviors among police officers, firefighters, EMTs, and paramedics. Clin. Psychol. Rev..

[25] Smith A., Roberts K. (2003). Interventions for post-traumatic stress disorder and psychological distress in emergency ambulance personnel: A review of the literature. Emerg. Med. J..

[26] Mildenhall J. (2012). Occupational stress, paramedic informal coping strategies: A review of the literature. J. Paramed. Pract..

[27] Nowakowska K., Jabłkowska-Górecka K., Borkowska A. (2009). Coping styles and burning out syndrome in the emergency medicine students and emergency medicine students working as emergency workers. Psychiatry Clin. Psychol..

[28] Ogińska-Bulik N., Kobylarczyk M. (2015). Relation between resiliency and post-traumatic growth in a group of paramedics: The mediating role of coping strategies. J. Occup. Med. Environ. Health.

[29] Almutairi M.N., Mahalli A.A.E. (2020). Burnout and coping methods among emergency medical services professionals. J. Multidiscip. Healthc..

[30] Boland L.L., Mink P.J., Kamrud J.W., Jeruzal J.N., Stevens A.C. (2019). Social support outside the workplace, coping styles, and burnout in a cohort of EMS providers from Minnesota. Workplace Health Saf..

[31] Beldon R., Garside J. (2022). Burnout in frontline ambulance staff. JPP.

[32] Nowakowska S., Wolniewicz Ł. (2017). Professional burnout among nurses and paramedics. Med. Sci. Pulse.

[33] Maslach C., Leiter M.P. (2016). Understanding the burnout experience: Recent research and its implications for psychiatry. World Psychiatry.

[34] Reardon M., Abrahams R., Thyer L., Simpson P. (2020). Prevalence of burnout in paramedics: A systematic review of prevalence studies. Emerg. Med. Australas..

[35] Department of Health (2019). Ministerstwo Zdrowia/Co Robimy/System Państwowe Ratownictwo Medyczne [Health Department/What We Do/Emergency Medical Services]. https://www.gov.pl/web/zdrowie/system-panstwowe-ratownictwo-medyczne.

[36] Santinello M. (2007). Link Burnout Questionnaire, Manual.

[37] Jaworowska A. (2014). Kwestionariusz Wypalenia Zawodowego Massimo Santinello. Polska Normalizacja [Massimo Santinello’s Burnout Questionnare. Polish Norms].

[38] Mańkowska B. (2018). Wypalenie zawodowe, dylematy wokół istoty zjawiska oraz jego pomiaru [Burnout: Dilemmas related to the concept and its measurement]. Pol. Forum Psychol..

[39] Sygit-Kowalkowska E., Weber-Rajek M., Herkt M., Ossowski R. (2017). Wypalenie zawodowe u funkcjonariuszy służby więziennej, rola osobowości i wybranych cech zawodowych [Occupational burnout in prison guards, the role of personality and selected occupational factors]. Med. Pr..

[40] Endler N.S., Parker J.D. (1990). Multidimensional assessment of coping: A critical evaluation. J. Pers. Soc. Psychol..

[41] Strelau J., Jaworowska A., Wrześniewski K., Szczepaniak P. (2005). Kwestionariusz Radzenia Sobie w Sytuacjach Stresowych CISS. Podręcznik do Polskiej Normalizacji [Coping Inventory for Stressful Situations. Polish Normalization Guide].

[42] Jabłońska A. (2015). Trafność teoretyczna Kwestionariusza Radzenia Sobie w Sytuacjach Stresowych CISS ze szczególnym uwzględnieniem trafności teoretycznej skali stylu skoncentrowanego na unikaniu [Theoretical validity of the Coping Inventory for Stressful Situations (CISS) with a special focus on the theoretical validity of the avoidance subscale]. Testy Psychol. Prakt. Bad..

[43] Kock N., Mayfield M. (2015). PLS-based SEM Algorithms: The good neighbor assumption, collinearity, and nonlinearity. Int. J. Inf. Manag..

[44] Garson G.D. (2016). Partial Least Squares: Regression & Structural Equation Models.

[45] Kock N. (2011). Using WarpPLS in e-collaboration studies: Mediating effects, control and second order variables, and algorithm choices. International Journal of e-Collaboration (IJeC).

[46] Kock N. (2018). Should bootstrapping be used in PLS-SEM? Toward stable *P*-Value Calculation methods. J. Appl. Struct. Equ. Model..

[47] Hamdan M., Hamra A.A. (2017). Burnout among workers in emergency Departments in Palestinian hospitals: Prevalence and associated factors. BMC Health Serv. Res..

[48] Williams B., Lau R., Thornton E., Olney L.S. (2017). The relationship between empathy and burnout—Lessons for paramedics: A scoping review. Psychol. Res. Behav. Manag..

[49] Bagherian F., Hosseini S.A. (2019). Burnout and job satisfaction in the emergency department staff: A review focusing on emergency physicians. Int. J. Med. Investig..

[50] Wang P., Wagner T.A., Boyar S.L., Corman S.A., McKinley R.B., Connerley M., Wu J. (2016). The relationship between organizational family support and burnout among women in the healthcare industry: Core self-evaluation as moderator. Handbook on Well-Being of Working Women. International Handbooks of Quality-of-Life.

[51] Donnelly E., Bradford P., Davis M., Hedges C., Klingel M. (2016). Predictors of posttraumatic stress and preferred sources of social support among Canadian paramedics. CJEM.

[52] Chou L.-P., Li C.-Y., Hu S.C. (2014). Job stress and burnout in hospital employees: Comparisons of different medical professions in a regional hospital in Taiwan. BMJ Open.

[53] Dubravka I., Nesek A.V., Srzić I., Stepić A., Pintaric H. (2017). Burnout syndrome in emergency medicine. Hong Kong J. Emerg. Med..

[54] Low Z.X., Yeo K.A., Sharma V.K., Leung G.K., McIntyre R.S., Guerrero A., Lu B., Sin Fai Lam C.C., Tran B.X., Nguyen L.H. (2019). Prevalence of burnout in medical and surgical residents: A meta-analysis. Int. J. Environ. Res. Public Health.

[55] Ortega-Campos E., Cañadas-De la Fuente G.A., Albendín-García L., Gómez-Urquiza J.L., Monsalve-Reyes C., de la Fuente-Solana E.I. (2019). A multicentre study of psychological variables and the prevalence of burnout among primary health care nurses. Int. J. Environ. Res. Public Health.

[56] McWilliams L.A., Cox B.J., Enns M.W. (2003). Use of the Coping Inventory for Stressful Situations in a clinically depressed sample: Factor structure, personality correlates, and prediction of distress. J. Clin. Psychol..

[57] Keller K.L., Koenig W.J. (1989). Management of stress and prevention of burnout in emergency physicians. Ann. Emerg. Med..

[58] Udemezue O.I. (2018). Burnout and psychiatric morbidity among doctors in the UK: A systematic literature review of prevalence and associated factors. Br. J. Psych. Bull..

[59] Xu H.G., Johnston A.N., Greenslade J.H., Wallis M., Elder E., Abraham L., Ogilvie T., Carlström E., Crilly J. (2019). Stressors and coping strategies of emergency department nurses and doctors: A cross-sectional study. Australas. Emerg. Care.

[60] Duschek S., Bair A., Haux S., Garrido A., Janka A. (2020). Stress in paramedics: Relationships with coping strategies and personality traits. Int. J. Emerg. Serv..

[61] Ghaniyoun A., Shakeri K., Heidari M. (2017). The association of psychological empowerment and job burnout in operational staff of tehran emergency center. Indian J. Crit. Care Med..

[62] Moukarzel A., Michelet P., Durand A.C., Sebbane M., Bourgeois S., Markarian T., Bompard C., Gentile S. (2019). Burnout syndrome among emergency department staff: Prevalence and associated factors. Biomed Res. Int..

[63] Zhang Y., Feng X. (2011). The relationship between job satisfaction, burnout, and turnover intention among physicians from urban state-owned medical institutions in Hubei, China. BMC Health Serv. Res..

[64] Jaiswal A., Singh T., Arya Y.K. (2020). “Psychological antibodies” to safeguard frontline healthcare warriors mental health against COVID-19 pandemic-related psychopathology. Front. Psychiatry.

[65] Pirkis J., John A., Shin S., Del Pozo-Banos M., Arya V., Analuisa-Aguilar P., Appleby L., Arensman E., Bantjes J., Baran A. (2021). Suicide trends in the early months of the COVID-19 pandemic: An interrupted time-series analysis of preliminary data from 21 countries. Lancet Psychiatry.

[66] Lin Z., Narayanan K., Suryadevara V., Teodorescu C., Reinier K., Uy-Evanado A., Chugh H., Arya V., Analuisa-Aguilar P., Appleby L. (2015). Occupation and risk of sudden death in a United States community: A case-control analysis. BMJ Open.

