# Supplementary material for: Work-Related Suicide Exposure, Occupational Burnout, and Coping in Emergency Medical Services Personnel in Poland
Source: Int J Environ Res Public Health. 2022 Jan 20;19(3):1156. doi: 10.3390/ijerph19031156 (PMC8835152; doi:10.3390/ijerph19031156)
Supplement: Supplementary file 1 [file ijerph-19-01156-s001.zip › ijerph-1483058-supplementary.pdf]

Witczak-Błoszyk et al., Work-related suicide exposure, occupational burnout and coping in Emergency Medical Services personnel in Poland. IJERPH, 2021.

Supplementary material

Suicide exposure, burnout and coping

Table S1: Model fit.

Coefficient	Value
AVIF	1,12
GoF	0,19
SPR	0,93
SSR	0,78
SRMR	0,12
SMAR	0,09
χ^2	53.42, p<0,001

AVIF = Average Variance Inflation Factor; GoF = Goodness of Fit; SPR = Simpson's Paradox Ratio; SSR = Statistical Suppression Ratio; SRMR = Standardized Root Mean Squared Residual; SMAR = Standardized Mean Absolute Residual; X^2 = Chi Square

Table S2: Measurement accuracy of the scales used and the explained measurement variance.

Measurement	R^2	ΔR^2	α	AVE
Psycho-physical exhaustion	0,02	0,01	0,70	0,38
Relational deterioration	0,21	0,20	0,66	0,37
Professional inefficacy	0,03	0,02	0,64	0,34
Disillusion	0,05	0,04	0,83	0,53
Task-oriented coping	0,04	0,03	0,87	0,36
Emotion-oriented coping	0,03	0,02	0,91	0,42
Avoidance coping	0,05	0,04	0,83	0,30
Distraction	0,03	0,03	1,00	1,00
Social diversion	0,04	0,03	1,00	1,00
Participation in suicide rescue	-	-	1,00	1,00
Time from the start of work	-	-	1,00	1,00
Number of rescue operations	-	-	1,00	1,00

Note. R^2= coefficient of determination; ΔR^2 = corrected R2; α = coefficient of reliability Cronbach's Alpha (desired coefficient value > = .75); AVE = Average Variance Extracted (desired coefficient value >= .50).

Access to professional psychological support and other workplace support programs, burnout, and coping

Table S3: Model fit.

Coefficient	Value
AVIF	1,75
GoF	0,12
SPR	0,78
SSR	0,85
SRMR	0,12
SMAR	0,09
χ^2	55.76, P<0.001

AVIF = Average Variance Inflation Factor; GoF = Goodness of Fit; SPR = Simpson's Paradox Ratio; SSR = Statistical Suppression Ratio; SRMR = Standardized Root Mean Squared Residual; SMAR = Standardized Mean Absolute Residual; X^2 = Chi Square

Table S4: Measurement accuracy of the scales used and the explained measurement variance.

Measurement	R^2	ΔR^2	α	AVE
Psycho-physical exhaustion	0,02	0,02	0,70	0,39
Relational deterioration	0,07	0,06	0,66	0,37
Professional inefficacy	0,00	0,01	0,64	0,34
Disillusion	0,04	0,03	0,83	0,53
Task-oriented coping	0,01	0,01	0,87	0,36
Emotion-oriented coping	0,01	0,00	0,91	0,42
Avoidance coping	0,01	0,01	0,83	0,30
Distraction	0,00	0,01	1,00	1,00
Social diversion	0,03	0,02	1,00	1,00
Is a psychologist employed there?	-	-	1,00	1,00
Can workers benefit from his/her competence and support?	-	-	1,00	1,00
Is there another support system at work?	-	-	1,00	1,00

R^2= coefficient of determination; ΔR^2 = corrected R2; α = coefficient of reliability Cronbach's Alpha; AVE = Average Variance Extracted.

Age, gender, years in employment, workload, burnout, and coping

Table S5: Model fit.

Coefficient	Value
AVIF	2,72
GoF	0,19
SPR	0,77
SSR	0,88
SRMR	0,12
SMAR	0,09
χ^2	56,732, p<0,001

AVIF = Average Variance Inflation Factor; GoF = Goodness of Fit; SPR = Simpson's Paradox Ratio; SSR = Statistical Suppression Ratio; SRMR = Standardized Root Mean Squared Residual; SMAR = Standardized Mean Absolute Residual; X^2 = Chi Square

Table S6: Measurement accuracy of the scales used and the explained measurement variance.

Measurement	R^2	ΔR^2	α	AVE
Psycho-physical exhaustion	0,02	0,01	0,70	0,38
Relational deterioration	0,10	0,08	0,66	0,37
Professional inefficacy	0,05	0,04	0,64	0,34
Disillusion	0,05	0,04	0,83	0,53
Task-oriented coping	0,03	0,02	0,87	0,36
Emotion-oriented coping	0,08	0,07	0,91	0,42
Avoidance coping	0,06	0,05	0,83	0,30
Distraction	0,04	0,03	1,00	1,00
Social diversion	0,05	0,03	1,00	1,00
Age	-	-	1,00	1,00
Gender	-	-	1,00	1,00
Place of residence size	-	-	1,00	1,00
Job seniority	-	-	1,00	1,00
Workload	-	-	1,00	1,00

Note. R^2= coefficient of determination; ΔR^2 = corrected R2; α = coefficient of reliability Cronbach's Alpha; AVE = Average Variance Extracted.
